# Comorbidity and age in the modelling of stroke: are we still failing to consider the characteristics of stroke patients?

**DOI:** 10.1136/bmjos-2019-100013

**Published:** 2020-02-24

**Authors:** Sarah K McCann, Catherine B Lawrence

**Affiliations:** 1 QUEST - Center for Transforming Biomedical Research, Berlin Institute of Health (BIH), Charité - Universitätsmedizin Berlin, Berlin, Germany; 2 Division of Neuroscience and Experimental Psychology and Lydia Becker Institute of Immunology and Inflammation, Faculty of Biology, Medicine and Health, Manchester Academic Health Science Centre, The University of Manchester, Manchester, UK

**Keywords:** stroke, comorbidity, ischaemia, aged, preclinical

## Abstract

Stroke is a significant cause of mortality and morbidity for which there are limited treatment options. Virtually all drug interventions that have been successful preclinically in experimental stroke have failed to translate to an effective treatment in the clinical setting. In this review, we examine one of the factors likely contributing to this lack of translation, the failure of preclinical studies to consider fully the advanced age and comorbidities (eg, hypertension or diabetes) present in most patients with stroke. Age and comorbidities affect the likelihood of suffering a stroke, disease progression and the response to treatment. Analysing data from preclinical systematic reviews of interventions for ischaemic stroke we show that only 11.4% of studies included an aged or comorbid model, with hypertension being the most frequent. The degree of protection (% reduction in infarct volume) varied depending on the comorbidity and the type of intervention. We consider reasons for the lack of attention to comorbid and aged animals in stroke research and discuss the value of testing a potential therapy in models representing a range of comorbidities that affect patients with stroke. These models can help establish any limits to a treatment’s efficacy and inform the design of clinical trials in appropriate patient populations.

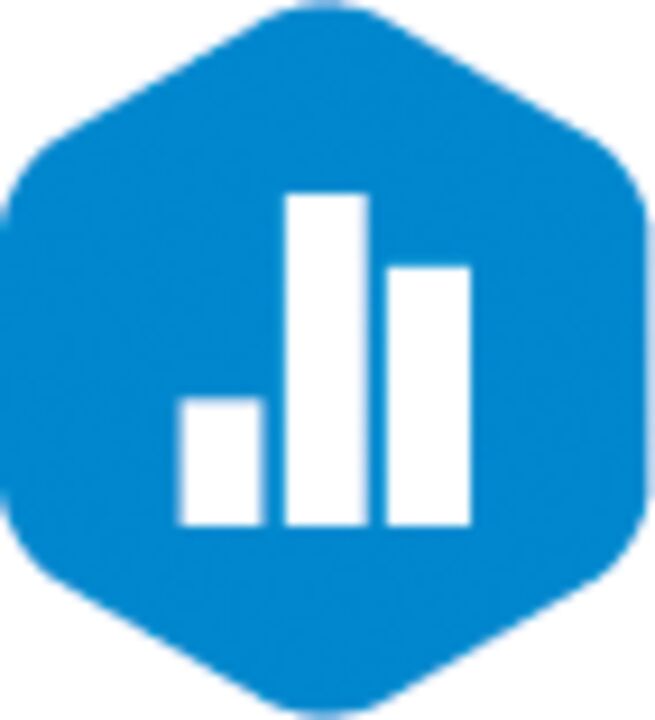

## Introduction

Stroke is a significant cause of mortality and the leading cause of neurological disability that negatively impacts long-term physical and mental health. Ischaemic stroke accounts for 85% of all strokes and is most commonly caused by occlusion of the middle cerebral artery. Treatment options for ischaemic stroke are currently limited to establishing reperfusion through thrombolysis (with recombinant tissue plasminogen activator (rtPA)) or mechanical thrombectomy. Over 1000 drugs have been tested in preclinical ischaemic stroke models but of those that progress to clinical trial, all but rtPA have failed to show benefit in patients.^1^ The reasons for this discrepancy have been debated extensively for many years but include weaknesses in preclinical experimental design, resulting in low internal validity.^2–4^ Questions have also been raised over the external validity of preclinical findings: to what extent can experimental inferences be generalised from animal models to other settings, including to human patients? To address these translational issues, the Stroke Treatment Academic Industry Roundtable (STAIR) initiative was established, first convening in 1999 and updating their preclinical recommendations in 2009.^5 6^ These recommendations include the design of randomised, blinded studies, as well as considering sex differences, replication in a second species and inclusion of comorbidity.^6^ In the 20 years since the first STAIR conference, further attempts have been made to improve the translation of preclinical stroke studies to patient benefit through numerous initiatives. Stroke-specific and more general guidelines to increase the quality of experimental design, practice and reporting include Stem Cell Therapies as an Emerging Paradigm in Stroke (STEPS),^7–9^ RIGOR,^10 11^ Ischaemia Models: Procedural Refinements of In Vivo Experiments (IMPROVE),^12^ and Animal Research: Reporting of In Vivo Experiments (ARRIVE).^13 14^ Changes to journal requirements and collaborative efforts to improve translation through multicentre preclinical stroke trials have also been implemented.^15–18^ The reported quality of stroke experiments has improved over this time, a development not observed in related fields.^3 4^ While there is still room for improvement, this positions the stroke field ahead of many others in relation to internal validity. However, we are yet to successfully translate positive preclinical results, suggesting the involvement of issues more complex than low study quality.

## Not all patients are equal

One important, and often overlooked, aspect of preclinical research is failure to consider the underlying health of patients with stroke. While not exclusively a disease of the elderly (25% of strokes occur in individuals under the age of 65), the risk of stroke increases significantly with age. Advancing age means individuals are more likely to suffer from other conditions including high blood pressure (hypertension), diabetes, atherosclerosis, coronary heart disease and obesity—conditions that are referred to as comorbidities. Comorbidity is defined as a medical condition that co-occurs with another in the same person and up to 90% of stroke risk can be attributed to 1 of 10 comorbidities.^19^ The majority of patients with stroke have at least one comorbidity, with hypertension being the most common,^20^ and often patients have multiple comorbidities (also known as multimorbidity).^21^

## Why should we consider comorbidities?

Many of the comorbidities seen in patients with stroke are independent risk factors for stroke. In addition to this increased risk of stroke, advanced age and comorbidities such as hypertension and diabetes can affect stroke progression, in most cases worsening outcome.^22–26^ Stroke outcome is also altered in preclinical models of age and comorbidity. For example, neurological impairment and recovery after experimental stroke is often worse in older animals compared with their younger counterparts.^27 28^ Hypertension and diabetes are very common in the elderly and both have been shown to worsen outcome in experimental stroke models.^27 29^ Obesity also worsens outcome after experimental stroke, with more ischaemic damage and greater blood–brain barrier breakdown observed in obese rodents compared with controls.^30–33^ Furthermore, cell death occurs much more rapidly in obese animals, which could have important implications for the treatment of obese patients with stroke in terms of how quickly treatments need to be given after stroke onset.^32^ Thus, the presence of a comorbidity, both clinically and in experimental animals, alters the pathophysiology of stroke.

Comorbidities can also alter response to treatment after stroke. This idea is supported by work on NXY-059, an antioxidant drug that reduced ischaemic damage in experimental studies, but failed to show efficacy when tested in patients with stroke.^34^ When the preclinical data were systematically reviewed and analysed, several issues were identified including that little or no activity was seen in animals that were hypertensive.^35 36^ However, in the final NXY-059 trial (Stroke Acute Ischemic NXY Treatment (SAINT II)) the majority (77%) of patients had a history of hypertension.^34^ Furthermore, in preclinical studies NXY-059 was not assessed for efficacy in any other comorbidity, or in aged animals, while the average age of patients was 69 years in the SAINT II trial. Establishing in preclinical experiments any limits to a treatment’s efficacy can help determine appropriate treatment populations and avoid clinical trials including patients who are unlikely to benefit or risk adverse outcomes.

**Figure 1:**
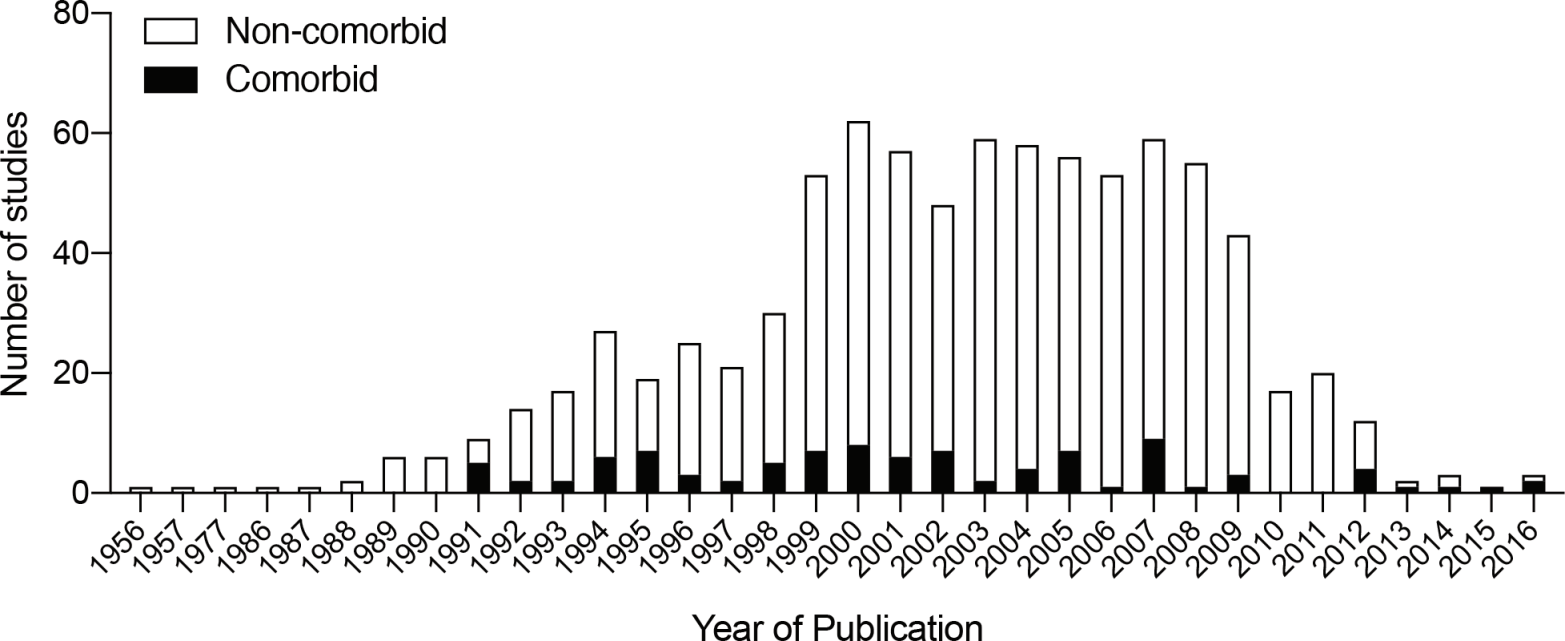
The number of included studies published per year. The number of publications reporting outcomes in aged or comorbid animals is shown in black and in non-comorbid animals in white.

**Figure 2:**
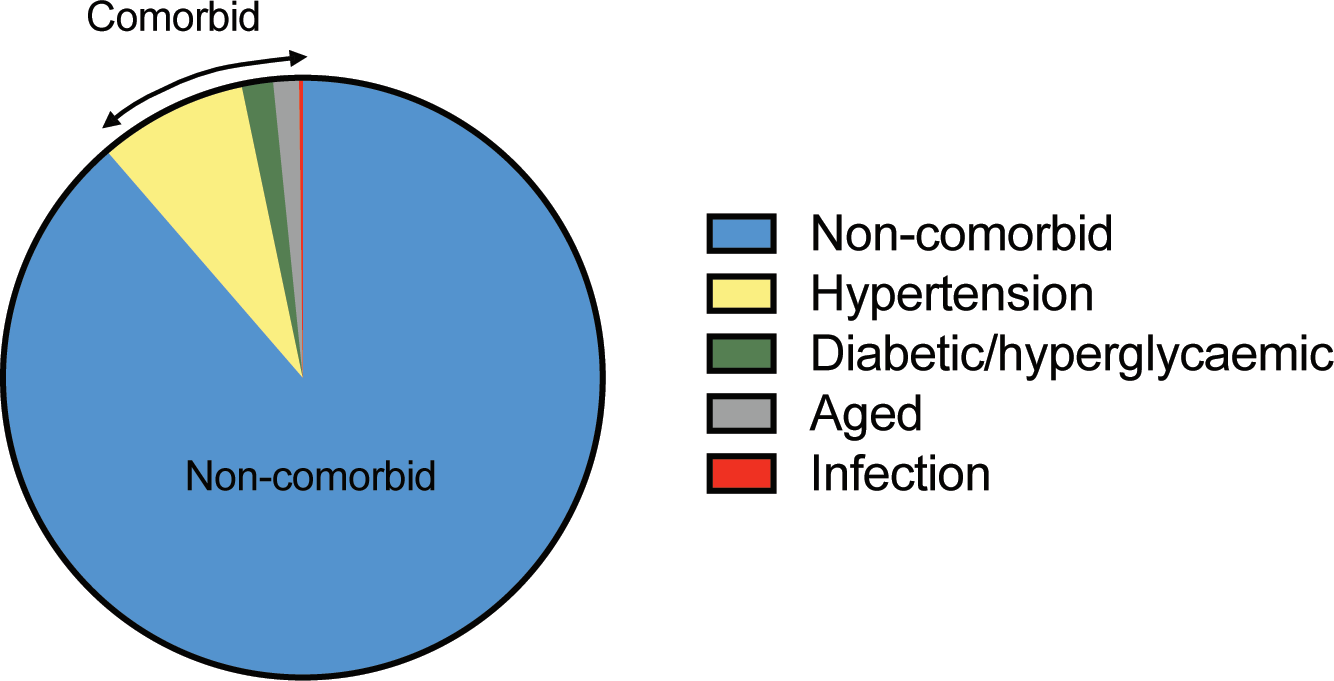
Inclusion of advanced age or a comorbidity in preclinical stroke studies testing an intervention. Presented as a percentage of all studies (n=842): n=746 non-comorbid (88.6%), n=68 hypertension (8.1%), n=14 diabetic/hyperglycaemic (1.7%), n=12 aged (1.4%), n=2 infection (0.2%).

**Figure 3:**
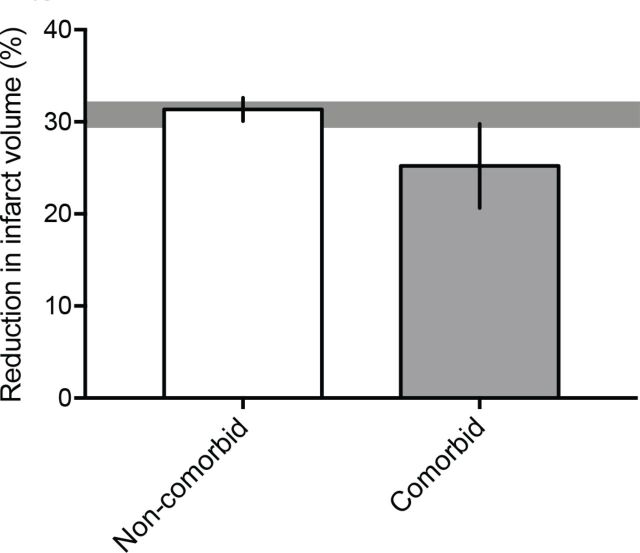
Reduction in infarct volume in non-comorbid versus comorbid animals. Less brain protection is observed after stroke and intervention in comorbid (n=163 outcomes) compared with non-comorbid (n=1703 outcomes) animals (p=0.003). Results are presented as the mean±95% CIs. The horizontal grey bar represents the 95% CIs of the combined effect of both comorbid and non-comorbid data.

**Figure 4:**
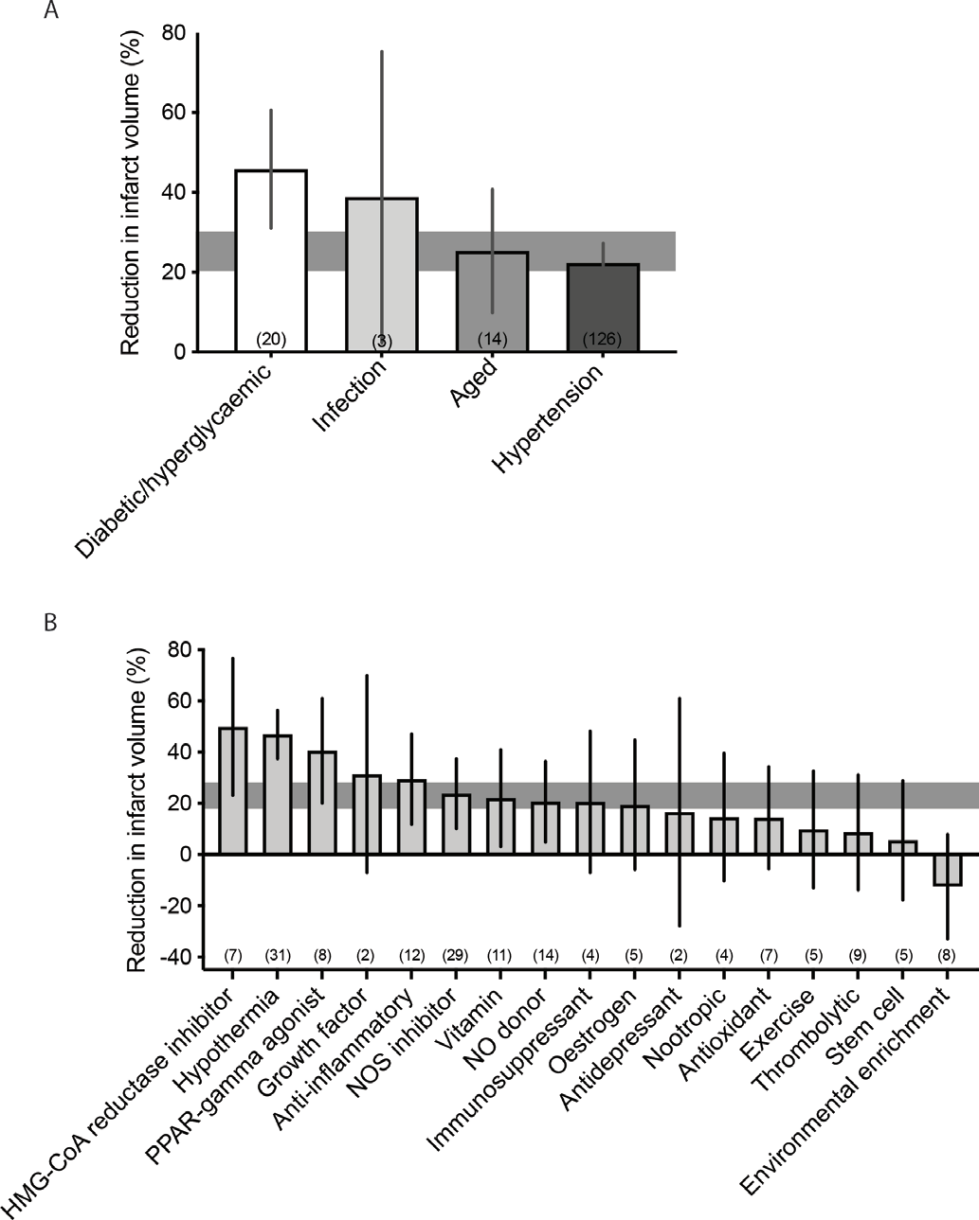
The impact of comorbidity and intervention type on reduction in infarct volume. The degree of protection observed in aged or comorbid animals varied depending on the type of comorbidity modelled (A; p=0.017) and the type of intervention administered (B; p<0.001). Results are presented as the mean±95% CIs, n=163 outcomes in total. The number of outcomes in each subgroup is shown in brackets. The horizontal grey bars represent the 95% CIs of the combined effect of the comorbid data. HMG-CoA, hydroxymethylglutaryl-coenzyme A; NO, nitric oxide; NOS, nitric oxide synthase; PPAR, peroxisome proliferator-activated receptor.

However, despite 10 years passing since the 2009 STAIR criteria recommended that ‘after initial evaluations in young, healthy male animals further studies should be performed in females, aged animals, and animals with co-morbid conditions such as hypertension, diabetes, and hypercholesterolemia’,^6^ the majority of preclinical studies to date continue to be performed in young and therefore ‘healthy’ non-comorbid animals. We analysed data from 25 preclinical systematic reviews of interventions for ischaemic stroke to investigate the frequency and impact of modelling advanced age and comorbidities (table 1). While this data set represents a non-random sample of publications and therefore may not be fully representative of modelling choices across the field, it is the largest currently available for preclinical stroke. Included studies were published from 1956 to 2016. The number of studies reporting outcomes in aged or comorbid models does not appear to increase over time, however, the number of included studies is low from 2010 onwards (figure 1). Outcomes were assessed in aged or comorbid animals in only 11.4% of all studies included in these systematic reviews (figure 2). The most commonly examined comorbidity was hypertension, which was reported in 8.1% of studies, with diabetes/hyperglycaemia next frequently studied (1.7%). Although advanced age is the major non-modifiable risk factor for stroke, only 1.4% of all included studies tested interventions in aged animals. Using meta-analysis, we assessed the impact of modelling these characteristics on the most commonly reported stroke outcome measure, infarct volume (table 1). Overall, treatment with any intervention reduced infarct volume by 30.8% (95% confidence interval (CI) 29.5% to 32.0%). However, less protection was observed in aged or comorbid animals (25.2%, 95% CI 20.7% to 29.8%) compared with non-comorbid animals (31.3%, 95% CI 30.1% to 32.6%; figure 3). The degree of protection in aged or comorbid animals varied depending on which comorbidity was modelled and the type of intervention administered (figure 4A, B). The least protection was observed in hypertensive animals (22.3% reduction in infarct volume; 95% CI 17.3% to 27.3%, 126 comparisons; figure 4A), the most common comorbidity in patients with stroke. Reduced intervention efficacy has also been shown in a previous review of data from hypertensive animals.^37^ In contrast, in the present data set, modelling diabetes or hyperglycaemia (figure 4A) appeared to result in a greater response to treatment than that seen in non-comorbid animals overall (figure 3). These data are limited (20 comparisons) and should be interpreted with caution; however, there is evidence for a greater improvement in diabetic versus non-diabetic animals following certain interventions.^38^ When we examined the efficacy of different intervention types, the most robust protection was provided by hypothermia (46.9% reduction in infarct volume; 95% CI 37.5% to 56.3%; figure 4B). However, for most of the intervention types tested there are insufficient data to determine if any effect is present in aged and comorbid animals.

**Table 1:** Data source and methods

Data characteristics	
Systematic reviews, n	25
Systematic reviews including aged or comorbid outcomes, n	22
Total number of studies included in systematic reviews	842
Studies with aged or comorbid outcomes, n	96 (11.4%)
Total number of outcomes included in systematic reviews	3187
Aged or comorbid outcomes, n	314 (9.9%)
Total number of infarct volume outcomes	1866
Aged or comorbid infarct volume outcomes, n	163 (8.7%)
Publication date of included studies	1956–2016

Data were drawn from the Collaborative Approach to Meta Analysis and Review of Animal Data from Experimental Studies (CAMARADES) Focal Ischaemic Stroke Database,^45^ a non-random sample of preclinical systematic reviews of interventions for ischaemic stroke. We analysed overall the frequency of modelling comorbidities in studies included in the database. We then assessed the impact of modelling advanced age or comorbidities on infarct volume outcomes. Normalised mean difference effect sizes, representing the percentage reduction in infarct volume after intervention (vs control), were combined using random effects meta-analysis with restricted maximum likelihood (REML) estimate of between-study variance. The impact of modelling advanced age or a comorbidity, the type of comorbidity and the type of intervention were assessed using univariate meta-regression.^46^

## Challenges to using comorbid models in experimental stroke

A number of related factors likely contribute to preclinical researchers’ failure to consider routinely the inclusion of aged or comorbid animals in their research. The addition of these animals can significantly increase experimental costs, as often they (eg, hypertensive, diabetic or obese animals) need to be purchased from specialised suppliers, or maintained for long periods (eg, on a particular diet) to induce a relevant phenotype. The latter point, therefore, significantly increases the time it takes to perform a set of experiments, which has a negative impact on productivity in the short term. There can also be issues when using aged or comorbid animals as they are technically more demanding and mortality is often higher, especially in older animals, which can increase the number of animals required to maintain appropriate power in a study. Together, these issues might make gaining funding problematic. There are funding schemes that are starting to address this concern, such as the Medical Research Council Developmental Pathway Funding Scheme in the UK, which will fund preclinical therapy development.

Obstacles also exist at the level of the individual researcher. The research metrics currently used to inform academic hiring, promotion and granting decisions primarily favour novelty and impact, which can make this type of translational work unattractive to researchers trying to advance their careers in a highly competitive environment. Initiatives such as the Declaration on Research Assessment (https://sfdora.org/)^39^ and the Leiden Manifesto for research metrics^40^ aim to address many of the issues associated with current research assessment practices. However, the manifold challenges associated with implementing the proposed reforms, including the coordinated effort required from different stakeholders, mean that it may take years to effect real change.

## Incorporating age and comorbidities in experimental stroke

Our analysis highlights differences in treatment effects depending on the comorbidity modelled, indicating that experiments in multiple models may be necessary to establish the efficacy profile of individual interventions. The altered pathology in comorbid stroke also supports modified profiles of potential therapeutic targets. The vast number of potential comorbidities and multimorbidity combinations in patients with stroke precludes testing candidate drugs under all possible conditions. However, we can focus on those most commonly present clinically, for example, advanced age and hypertension. The potential for multiple common diseases (eg, diabetes and obesity) to interact and modify underlying mechanisms and response to treatment should also be considered. Detailed recommendations have been made regarding model selection and experimental design for preclinical stroke studies modelling hypertension and diabetes, two of the most common comorbidities.^41^

It is important to separate modelling acute changes in blood pressure or glucose levels from chronic comorbidity with related pathophysiological alterations, for example, changes in the inflammatory response, immune modulation and vasculature. Additionally, whether a comorbidity is controlled or uncontrolled at the time of stroke may influence outcome. Medications are routinely used to control chronic comorbidity or multimorbidity in patients, a variable rarely modelled preclinically. Numerous interventions are available for the long-term management of comorbidities such as hypertension or high cholesterol, creating the possibility of drug–drug interactions that alter the efficacy of potential stroke therapies. Pooled systematic review data revealed that antihypertensive agents have primarily been investigated in animal models of stroke for the acute management of hypertension, rather than coadministered with a candidate neuroprotectant to test for possible interactions.^37^ Incorporating these clinically relevant factors can increase experimental design complexity severalfold. The most important studies to focus on initially involve the most common clinical scenarios and the potential for adverse pharmaceutical interactions based on in vitro testing.

Exploratory research investigating potential new therapeutic targets or providing initial efficacy data need not involve experiments in an extensive range of aged and comorbid models. It is however imperative that confirmatory studies, carried out to inform candidate suitability for clinical assessment, assess efficacy in models representative of the intended patient populations.^42^ Multicentre preclinical trials have been proposed as a means to robustly assess the efficacy of highly promising preclinical candidate interventions before they advance to clinical testing.^16^ Incorporating aged and comorbid models into this framework would remove the onus from individual groups to perform multiple costly experiments and provide cross-validation of results across a network of laboratories.

When designing early-stage clinical trials, limiting treatment to populations where preclinical efficacy has been demonstrated, while reducing the number of eligible patients, would increase the rigour of the translational process.^9^ Indeed, one of the proposed reasons for translational failure is recruitment of patients outside the demonstrated preclinical efficacy profile.^16^ Incorporating aged and comorbid animal models at the appropriate stage of drug development will increase scientific value and clinical relevance, resulting in more ethical animal research.^43 44^

## Summary

It is now widely agreed within the preclinical stroke research field that important patient characteristics including advanced age and comorbidities should be considered in experimental stroke. However, data from 25 preclinical systematic reviews of interventions for ischaemic stroke indicate that we are still failing to consider the underlying health of stroke patients. Less than 12% of studies assessed outcome in an aged or comorbid model and, when compared with non-comorbid models, a relative reduction in treatment efficacy of 20% is seen in these animals overall. Efficacy varied depending on the individual intervention tested and across the different comorbidities, indicating that it may be necessary to assess new treatments in a range of models representing different patient populations, before they are considered for clinical trial. The current failure to model relevant patient characteristics, despite their relevance in the context of stroke research and their impact on intervention efficacy, is likely occurring due to multiple related factors involving stakeholders including funders, research institutes and researchers. Moving forward, by addressing these factors and ensuring concordance between the characteristics of patients with stroke and experimental models, the external validity of findings from animal models will improve, increasing the likelihood of successful translation to clinical benefit.
